# Association of health locus of control with anxiety and depression and mediating roles of health risk behaviors among college students

**DOI:** 10.1038/s41598-025-91522-x

**Published:** 2025-03-04

**Authors:** Wenzhen Li, Zhiya Zhao, Dajie Chen, Mei-Po Kwan, Lap Ah Tse

**Affiliations:** 1 Jockey Club School of Public Health and Primary Care of the Chinese University of Hong Kong, Hong Kong SAR, China; 2 Shenzhen Research Institute of the Chinese University of Hong Kong, Shenzhen, China; 3 Department of Social Medicine and Health Management, School of Public Health, Tongji Medical College, Huazhong University of Science and Technology, Wuhan, 430030 Hubei China; 4 Department of Health Services and Management, Wuhan Polytechnic University, Wuhan, 430030 Hubei China; 5 Department of Geography and Resource Management, The Chinese University of Hong Kong, Hong Kong SAR, China; 6 Institute of Space and Earth Information Science, The Chinese University of Hong Kong, Hong Kong SAR, China

**Keywords:** Health locus of control, Health risk behavior, Anxiety, Depression, Risk factors, Psychology, Human behaviour

## Abstract

We aimed to assess the association of health locus of control with anxiety and depression, and explore the mediating effects of health risk behaviors. A multi-stage cluster random sampling method was used among Chinese college students. Logistic regression models were used to explore the associations of health locus of control with anxiety and depression. Structural equation models were used to explore the mediation roles of health risk behaviors in the associations of health locus of control with anxiety and depression. A total of 3,951 college students were included in this study. Internality was associated with lower prevalence of depression (OR = 0.94, 95% CI, 0.91–0.97), powerful others externality was also associated with lower prevalence of anxiety and depression (0.92, 0.88–0.96; 0.93, 0.89–0.96), while chance externality was associated with higher risk of anxiety and depression (1.13, 1.08–1.18; 1.24, 1.20–1.28). The mediated proportion of health risk behaviors in associations of internality, powerful others externality, chance externality with anxiety was 7.55%, 2.37% and 2.18%, respectively. The mediated proportion of health risk behaviors in associations of powerful others externality, chance externality with depression was 10.48% and 2.14%, respectively. Health locus of control is associated with anxiety and depression that are mediated by health risk behaviors.

## Introduction

Rapid demands on advanced technology in modern society have posed the college students increasing study pressure that exacerbates their mental health problems^1^. Academic pressure, lifestyle, and employment competition are the common factors associated with anxiety and depression among college students^2–4^. The COVID-19 epidemic has created more resistance for college and the superposition of many unfavorable conditions makes the psychological pressure of college students greater than ever before. Previous studies have showed high prevalence of depression and anxiety among college students^5^, especially during and after COVID-19 epidemic^6–8^. To date, a large amount of influencing factors of depression and anxiety have been explored to identify the key factors, however, individual’s cognitive style and attribution tendency towards their own health status are very important for mental health^9^, and it is unclear how they impact college students’ mental health.

### Theoretical background and hypothesis development

Health locus of control (HLC), as an important psychological factor, has been widely used in psychological studies^10,11^ including among college students^12^. HLC was first proposed by American scholar Roger in his Social Learning and Clinical Psychology Theory in the 1950s^13^. Rotter defines the source of control as what person or thing an individual feel is responsible for his or her life events, behavior results, health, etc. HLC was developed by Wallston ^14,15^ and finally it has three dimensions - internality, powerful others externality and chance externality. According to the Health Locus of Control Theory^16^, individuals’ health-related behaviors are associated with their perception of their ability to overcome health problems. The internality dimension represents the degree of confidence for the individuals to control their own health outcomes, and has been found to be linked to engage in positive and protective health behaviors^17^. The powerful others externality dimension represents the individual’s trust in others’ (friends, relatives, family members, teachers, etc., especially health professionals) ability to control their own health outcomes, which may link with better will-being by alleviating self-burden and higher sense of happiness resulted from getting effective others’ help^18^. And the chance externality dimension represents the extent to which individuals trust non-human factors (such as chance, luck, etc.) to determine their own health outcomes. A person with external externality showed a lower self-efficacy for one’s own health, which may lead to worse health outcomes^19^.

Previous studies have shown that health locus of control was associate with anxiety and depression^20,21^, but the results were still controversial to be confirmed further. Furthermore, research on the relationship between health locus of control and anxiety and depression in China is limited, while the research focusing on college students is even less. In addition, health risk behaviors including smoking, drinking, low physical activity, long screen time, and unhealthy diet behavior were also found to be associated with health locus of control^22–24^, some studies^24,25^ have indicated that HLC is an antecedent influencing factor of development of healthy lifestyle manifested by health risk behaviors^26^. Chance externality has been found to be directly related to unhealthy behaviors such as smoking, alcohol consumption and unhealthy dieting^27^, while powerful others externality was negatively associated with health risk behaviors in individualistic cultural backgrounds^24^; however, in collectivist cultures, this relationship might be the opposite^28^ and thus, it needs to be further investigated and determined.

Furthermore, health risk behaviors also linked to anxiety and depression^29–31^, a large number of studies has showed the significant associations between physical exercise and anxiety^32–34^ and depression^35^, unhealthy diet and mental health^36,37^, other unhealthy behaviors and mental health^38,39^; however, the mediating role of health risk behaviors, which were considered as a whole variate in the association of HLC with anxiety and depression is still unknown.

Thus, we put forward the following two hypotheses:

*Hypothesis H1* health locus of control is associated with anxiety and depression, and individuals with higher of internal and powerful others externality will have lower levels of anxiety and depression, while that with higher of chance externality will have higher levels of anxiety and depression.

*Hypothesis H2* Health risk behaviors may play a mediating role of in the association of HLC with anxiety and depression.

**Fig. 1:**
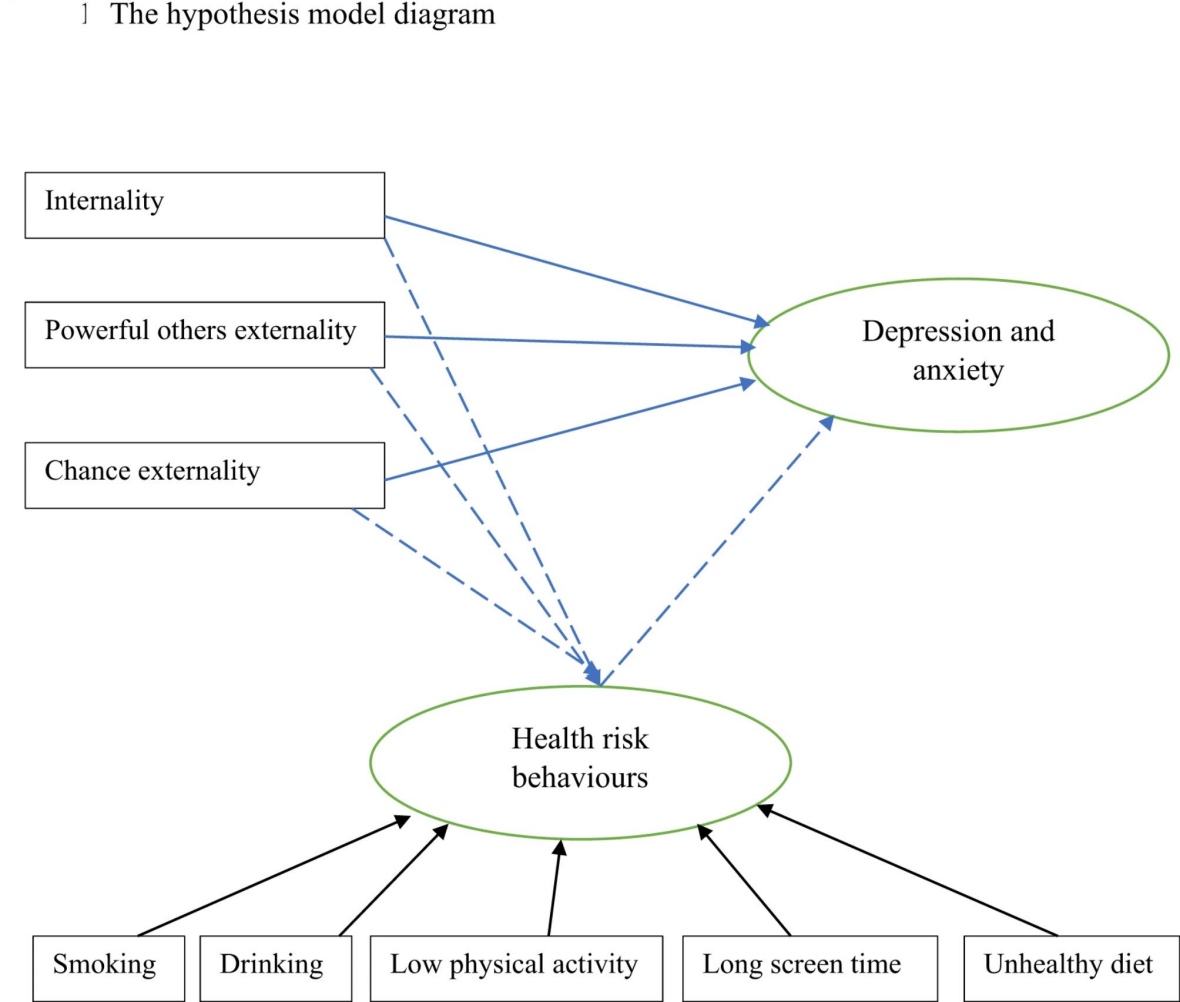
The hypothesis model diagram.

We thus conducted this study to examine the hypothesis in 3,951 Chinese college students, and proposed research hypothesis model is showed in Fig. 1. Our study will provide some evidence that assessing individual’ HLC can provide insights into the specific beliefs and attitudes that may impact their ability to manage their health, as well as the strategies that they may use to promote their own health.

## Results

### Basic characteristics

The baseline characteristics of the eligible college students according to anxiety and depression are shown in Table 1. We found a significant difference of anxiety among major characteristics, study score, and study burden. Meanwhile, we also found a significant difference of depression among students with study score, and study burden.

**Table 1:** Sociodemographic characteristics of participants by anxiety and depression (*n* = 3951).

Variables	Anxiety			Depression		
	*n* (%)	χ^2^	*P*	*n* (%)	χ^2^	*P*
Gender		1.30	0.25		0.79	0.37
Male	892 (53.3)			980 (58.5)		
Female	1255 (55.1)			1365 (60.0)		
Major		7.41	0.01		2.87	0.09
Medical	630 (51.1)			707 (57.4)		
Non-medical	1517 (55.8)			1638 (60.2)		
Only child in the family		1.80	0.18		0.05	0.83
Yes	759 (55.8)			804 (59.1)		
No	1388 (53.6)			1541 (59.5)		
Father’s education level		1.79	0.62		2.48	0.48
Primary school and below	372 (53.7)			415 (59.9)		
Junior high school	847 (54.4)			939 (60.3)		
Senior high school	485 (53.1)			522 (57.2)		
College degree or above	443 (56.2)			469 (59.5)		
Mother’s education level		3.11	0.38		0.82	0.85
Primary school and below	617 (54.7)			665 (58.9)		
Junior high school	760 (52.6)			870 (60.3)		
Senior high school	436 (55.6)			463 (59.1)		
College degree or above	334 (56.2)			347 (58.4)		
Self-rated family economic conditions (RMB)	3.05	0.55		3.48	0.48	
0-999	272 (53.4)			314 (61.7)		
1000–1999	356 (54.9)			381 (58.7)		
2000–2999	856 (53.8)			925 (58.1)		
3000–4999	386 (57.1)			415 (61.4)		
>=5000	277 (52.8)			310 (59.1)		
Average monthly consumption (RMB)		6.92	0.07		5.56	0.14
<=1000	703 (53.3)			791 (60.0)		
1001–1500	878 (53.2)			949 (57.5)		
1501–2000	384 (56.8)			412 (61.0)		
> 2000	182 (59.9)			193 (63.5)		
Study score		18.27	< 0.001		28.8	< 0.001
Poor	321 (60.6)			371 (70.0)		
Medium	1339 (51.9)			1489 (57.8)		
Good	487 (57.8)			485 (57.5)		
Study burden		140.54	< 0.001		68.30	< 0.001
Heavy	989 (66.0)			1013 (67.6)		
Medium	1100 (48.0)			1249 (54.5)		
Light	58 (36.0)			83 (51.6)		

### Association of health locus of control with anxiety and depression

The association of health locus of control with anxiety and depression is presented in Table 2. As to anxiety, internality (OR = 0.96, 95% CI, 0.95–0.98) and powerful others externality (OR = 0.95, 95% CI, 0.94–0.98) were associated with lower prevalence of anxiety, while chance externality was associated with higher prevalence of anxiety (OR = 1.12, 95%CI, 1.10–1.14). The results were similar to depression. Internality (OR = 0.96, 95%CI, 0.95–0.98) and powerful others externality (OR = 0.95, 95%CI, 0.93–0.97) were associated with lower prevalence of depression, while chance externality (OR = 1.11, 95%CI, 1.09–1.13) was associated with higher prevalence of depression.

**Table 2:** Association of health locus of control, health risk behaviors with anxiety and depression among college students.

	Anxiety			Depression		
	OR	95% CI	*P*	OR	95% CI	*P*
Health locus of control						
Internality	0.96	0.95–0.98	< 0.001	0.96	0.95–0.98	< 0.001
Powerful others externality	0.95	0.94–0.98	< 0.001	0.95	0.93–0.97	< 0.001
Chance externality	1.12	1.10–1.14	< 0.001	1.11	1.09–1.13	< 0.001
Health risk behaviors						
Smoking	1.41	1.04–1.93	0.029	1.20	0.88–1.64	0.25
Drinking	1.07	0.87–1.31	0.520	1.13	0.92–1.39	0.26
Low physical activity	1.30	1.12–1.53	< 0.001	1.31	1.12–1.53	< 0.001
Long screen time	1.08	0.84–1.39	0.532	1.21	0.95–1.56	0.13
Unhealthy diet behavior	1.35	1.16–1.56	< 0.001	1.56	1.35–1.81	< 0.001
Health risk behaviors	1.05	1.03–1.07	0.043	1.54	1.07–2.02	< 0.001

Adjusted for gender, major, only child in the family (yes, no), father’s education level, mother’s education level, self-rated family economic conditions, average monthly consumption (RMB), study score, study burden.

### Mediating role of health risk behaviors in the association of health locus of control with anxiety and depression

As to the association between health locus of control and health risk behaviors, internality (OR = 0.85, 95% CI, 0.80–0.93) and powerful others externality (OR = 0.96, 95% CI, 0.95–0.98) were associated with lower prevalence of health risk behaviors, while chance externality was associated with higher prevalence of health risk behaviors (OR = 1.03, 95%CI, 1.00-1.06). Association of health risk behaviors with anxiety and depression among college students is shown in Table 2. Health risk behaviors were both associated with higher prevalence of anxiety (OR = 1.05, 95%CI, 1.03–1.07) and depression (OR = 1.54, 95%CI, 1.07–2.02).

The mediating role of health risk behaviors in the association of health locus of control with anxiety and depression are shown in Table 3. The mediated proportion of health risk behaviors in the association of internality, powerful others externality, chance externality with anxiety was 7.6%, 2.4% and 2.2%, respectively. The mediated proportion of health risk behaviors in the association of powerful others externality, chance externality with depression was 10.5% and 2.1%, respectively.

**Table 3:** Mediating role of health risk behaviors in the association of health locus of control with anxiety and depression.

Variable	Effect	Dependent Variable						Mediated proportion, %
		Health risk behaviors			Anxiety			
		ß	95%CI	*P*	ß	95%CI	*P*	
Internality	Direct effect	-0.05	-0.09, -0.001	0.05	-0.05	-0.10, 0.003	0.06	7.6
	Indirect effect	-	-	-	-0.004	-0.01, -0.001	0.03	
	Total effect	-0.05	-0.09, -0.001	0.05	-0.05	-0.10, -0.002	0.05	
Powerful others externality	Direct effect	-0.08	-0.16, -0.01	< 0.001	-0.33	-0.41, -0.25	< 0.001	2.4
	Indirect effect	-	-	-	-0.01	-0.02, -0.002	0.02	
	Total effect	-0.08	-0.16, -0.01	< 0.001	-0.34	-0.42, -0.26	< 0.001	
Chance externality	Direct effect	0.12	0.06, 0.18	< 0.001	0.49	0.43, 0.56	< 0.001	2.2
	Indirect effect	-	-	-	0.01	0.01, 0.02	< 0.001	
	Total effect	0.12	0.06, 0.18	< 0.001	0.50	0.44, 0.57	< 0.001	
Health risk behaviors	Direct effect	-	-	-	0.09	0.06, 0.13	< 0.001	-
	Indirect effect	-	-	-	-	-	-	
	Total effect	-	-	-	0.09	0.06, 0.13	< 0.001	
		Health risk behaviors			Depression			
Internality	Direct effect	-0.04	-0.08, 0.003	0.07	-0.05	-0.10, -0.001	0.05	-
	Indirect effect	-	-	-	-0.004	-0.01, 0.001	0.07	
	Total effect	-0.04	-0.08, 0.003	0.07	-0.05	-0.10, -0.004	0.03	
Powerful others externality	Direct effect	-0.11	-0.19, -0.02	0.01	-0.46	-0.55, -0.37	< 0.001	10.5
	Indirect effect	-	-	-	-0.01	-0.02, -0.003	< 0.001	
	Total effect	-0.11	-0.19, -0.02	0.01	-0.47	-0.11, -0.38	< 0.001	
Chance externality	Direct effect	0.14	0.07, 0.21	< 0.001	0.64	0.56, 0.73	< 0.001	2.1
	Indirect effect	-	-	-	0.01	0.01, 0.02	< 0.001	
	Total effect	0.14	0.07, 0.21	< 0.001	0.66	0.57, 0.75	< 0.001	
Health risk behaviors	Direct effect	-	-	-	0.10	0.07, 0.14	< 0.001	-
	Indirect effect	-	-	-	-	-	-	
	Total effect	-	-	-	0.10	0.07, 0.14	< 0.001	

Adjusted for gender, major, only child in the family (yes, no), father’s education level, mother’s education level, self-rated family economic conditions, average monthly consumption (RMB), study score, study burden.

## Discussion

A series of strict prevention and isolation measures for a long time during the epidemic after sudden outbreak of COVID-19 has brought an immeasurable impact on the normal lives and study of college students, and the impact on their mental health cannot be ignored and psychological factors related depression and anxiety should be issued.

In the present study, we found that internality and powerful others externality were associated with lower prevalence of anxiety and depression, and the results for internality and chance externality were consistent with other publications^40^. College students with higher scores of internality are more likely to attribute the success to their own ability and effort, believe that they can control their own life, so they will experience more control over life, and thus have a positive effect on their emotions, making them not prone to be anxiety and depression^41^. With regard to powerful others externality, the results were controversial, our study showed that both depression and anxiety were associated with powerful others externality, however, a previous study suggested that depression was significantly moderated by powerful others externality but not for anxiety symptom^42^, and Sigurvinsdottir et al.’ study also showed the similar result^43^. The relationship between powerful others externality and anxiety should be further examined in the future. Besides, another study^40^ showed that powerful others externality were associated with higher depression score, one possible reason is that overreliance on external individuals to control one’s own health may fall into extreme helplessness and lead to poor mental health once these external forces go wrong, therefore, this relation needs to be further examined. The contradictory results suggested that evaluation of degree of powerful others externality should be paid more attention in targeting population and intervention practice. In addition, chance externality was associated with higher prevalence of anxiety and depression in our study. People who think that opportunities can have a greater impact on their life usually believe that the success or failure is due to luck and fate. Therefore, they are pessimistic and passive towards life, and gradually feel that they have lost control of life and feel powerless. This feeling of powerlessness will lead to anxiety and depression^44^. Our result was not completely consistent with a previous study, in which chance externality was significantly associated with depression, however, did not associated with anxiety^42^, and Khumalo et al.’ study^40^ also revealed a positive association between chance externality and depression. All these inconsistent results may result from differences in cultural meaning of perceived control, a meta-analysis study^45^ showed that the relationship between HLC and psychological symptoms differed among cultures with distinct individualist orientations, and external HLC does not carry the same negative connotations across cultures or population. All in all, the findings of our study have important public health implications for college students by identifying HLC as an important pathway in the development of psychological status, and focusing on the HLC may be crucial in enhancing the psychological well-being of college students, improving their self-efficacy and future interventions. And interventions should target students with lower internality or higher chance externality in the future.

Furthermore, we also explored the role of health risk behaviors in the association of health locus of control with anxiety and depression, and found that health risk behaviors mediated the association of all dimensions of health locus of control with anxiety, and mediated the association between powerful others externality, chance externality and depression, but not for internality and depression. It’s might be that more depressed individuals exhibit lower levels of internality and engage in risk health behaviors, and the reverse causality explanation needs to be examined in the future study with cohort design.

The mechanism underlying the association of HLC with anxiety and depression is unclear. We explored the role of health risk behaviors in the association of health locus of control with anxiety and depression. Consistent with previous studies, we found that health risk behaviors were not only associated with health locus of control^46,47^, but also with anxiety and depression. And health risk behaviors mediated the association of health locus of control with anxiety and depression^48,49^. The results indicated that the prevalence of anxiety and depression could be decreased by reducing the prevalence of health risk behaviors among college students with internality and powerful others externality. While the prevalence of anxiety and depression could be increased by increasing the prevalence of health risk behaviors among college students with chance externality. The specific mechanisms that underlie the association between these variables is still a need to further investigate in order to identify key variables that promote their psychological well-being and provide protective effects against mental health issues.

Our study has several major strengths. First, we collected the data from east, central and west China with a large simple size, which could increase the representability of the sample. Second, health locus of control, anxiety, and depression was assessed with MHLC, GAD-7 and PHQ-9 questionnaire, respectively. All these could contribute to the reliability of the results. However, some limitations should also be acknowledged. First, it is a cross-sectional study, which could not get the causality relationship. Second, our findings may be only representative of the Chinese college students, and the generalizability to other populations should be done with caution.

Our study shows that health locus of control is associate with anxiety and depression in college students, and health risk behavior plays a mediating role in the above-mentioned relationship. Effective intervention measures such as change of lifestyle should be implemented to prevent the occurrence of anxiety and depression among college students in the post-epidemic era of COVID-19.

## Methods

### Study population

A multi-stage cluster random sampling was used to conduct this study among Chinese college students from October to December 2020, which has been described in the previous publication^50^. Briefly, every two provinces were randomly selected from East, Central and West China, and then two universities were randomly selected from each province. All students in the selected universities were included to fill out an electronic questionnaire. The information includes the basic demographic and social psychological characteristics of the college students. The basic demographic characteristics include gender, type of school, grade, learning burden, etc. The social psychological characteristics included health locus of control, anxiety and depression. Finally, a total of 4102 online questionnaires were collected. After excluding 151 incomplete questionnaires, 3951 eligible questionnaires were retained in this study.

The study was approved by the Research Ethics Committee in Tongji Medical College, Huazhong University of Science and Technology, Wuhan, China. We confirm that all methods were carried out in accordance with relevant guidelines and regulations. The reporting of this study conforms to the Strengthening the Reporting of Observational Studies in Epidemiology (STROBE) statement, and informed consent was obtained from all subjects.

### Assessment of health locus of control

The Multidimensional Health locus of Control Questionnaire (MHLC) developed by Wallston et al.^14,15^ was used to assess health locus of control, which includes three dimensions: internality, powerful others externality and chance externality. Each dimension has 6 entries, totaling 18 entries. Likert 6 is used for scoring, and the answer range is from 1 point (very disapproval) to 6 points (very approval). The score range of each dimension is 6 ~ 36 points, and the total score range is 36 ~ 108 points. The higher the score, the stronger the tendency of the subject in this dimension.

### Assessment of anxiety

We adopted the generalized anxiety disorder 7-item scale (GAD-7) developed by Spitzer et al.^51^. The scale contains 7 items, and each item corresponds to one anxiety symptom, including anxious, not being able to stop worrying, worrying too much, hard to relaxing, easy to be annoyed, feeling something terrible will happen and restless. The answer ranges from 0 (none) to 3 (almost every day). The score ranges from 0 to 21. The score of 5, 10 and 15 represents the cut-off values of “mild”, “moderate” and “severe” anxiety, respectively.

### Assessment of depression

The 9-question depression scale from the patient health questionnaire (PHQ-9) developed by Spitzer et al.^52^ was used to assess depression. The scale contains 9 items, and each item corresponds to one depressive symptom, including loss of pleasure, depression, sleep disorder, lack of energy, eating disorder, low self-evaluation, difficulty in focusing fidgety and negative ideas. The answer ranges from 0 (no) to 3 (almost every day), and the score range is 0 to 27. The score of 5, 10, 15, and 20 represents the cut-off values of “mild”, “moderate”, “moderate-severe”, and “severe” depression, respectively.

### Health risk behaviors

Health risk behaviors were defined according to Chinese Adolescent Health-related Behavior Questionnaire (University Edition), including five dimensions: smoking, drinking, physical activity, screen time, and diet behavior. Smoking was defined as those who smoked at least one cigarette per day for more than half a year and smoked in the past 30 days. Drinking was defined as those who drank at least one time in the past 30 days. Long screen time was defined as the average time looking for computer or phone was more than 2 h per day. Physical activity was evaluated according to Physical Activity Rank Scale-3^53,54^, and physical activity volume = intensity × time × frequency, scoring range from 0 to 100 points, with scores ≤ 19 points, 20–42 points, and ≥ 43 points corresponding to low, medium, and high physical activity levels. In the present study, a score of ≤ 19 points is considered insufficient physical activity. Unhealthy diet behavior was defined as at least one behavior including often drink drinks, often eat dessert, often eat western fast food, do not drink milk, do not eat breakfast, partial food, unhealthy weight loss behavior.

### Statistical analysis

The sociodemographic characteristics of the participants were reported as the mean (SD) for continuous variables and as numbers (percentages) for categorical variables. Chi square test was used to compare the differences of anxiety and depression status quo under different demographic characteristics. Logistic regression models were used to explore the association of health locus of control with health risk behaviors, and anxiety and depression among college students. Meanwhile, structural equation models were used to explore the mediation roles of health risk behaviors in the association of health locus of control with anxiety and depression. All statistical analyses were performed using SPSS 25.0 and AMOS 26.0. The statistical tests were two-sided, and significance was set at *P <* 0.05.

## Data Availability

Data will be made available on request (Wenzhen Li, wenzhenli@cuhk.edu.hk).
